# Restoring the Secretory Function of Irradiation-Damaged Salivary Gland by Administrating Deferoxamine in Mice

**DOI:** 10.1371/journal.pone.0113721

**Published:** 2014-11-26

**Authors:** Junye Zhang, Lei Cui, Minhua Xu, Yuanli Zheng

**Affiliations:** 1 Stomatology Special Consultation Clinic, Ninth People’s Hospital, Shanghai Jiao Tong University, School of Medicine, Shanghai Key Laboratory of Stomatology, Shanghai, People’s Republic of China; 2 Department of Stomatology, Branch of Shanghai First People’s Hospital, Shanghai, People’s Republic of China; 3 Department of Plastic and Reconstructive Surgery, Ninth People’s Hospital, Shanghai Jiao Tong University, School of Medicine, Shanghai, People’s Republic of China; National Institutes of Health, United States of America

## Abstract

**Objectives:**

One of the major side effects of radiotherapy for treatments of the head and neck cancer is the radiation-induced dysfunction of salivary glands. The aim of the present study is to investigate the efficacy of deferoxamine (DFO) to restore the secretory function of radiation-damaged salivary glands in mice.

**Methods:**

DFO (50 mg/kg/d) was administered intraperitoneally in C_57_BL/6 mice for 3 days before and/or after point-fixed irradiation (18 Gy) of submandibular glands. The total 55 mice were randomly divided into: (1) Normal group: mice received no treatment (n = 5); (2) Irradiation group (IR): mice only received irradiation (n = 5); (3) Pre-DFO group (D+IR) (n = 10); (4) Pre+Post DFO group (D+IR+D) (n = 10); (5) Post-DFO group (IR+D) (n = 10); (6) For each DFO-treated group, the mice were intraperitoneally injected with 0.1 ml sterilized water alone (by which DFO was dissolved) for 3 days before and/or after irradiation, and served as control. Sham1: Pre-sterilized water group (n = 5); sham2: Pre+Post sterilized water group (n = 5); sham3: Post-sterilized water group (n = 5). The salivary flow rate (SFR) was assessed at 30^th^, 60^th^ and 90^th^ day after irradiation, respectively. After 90 days, all mice were sacrificed and their submandibular glands were removed for further examinations.

**Results:**

The salivary glands showed remarkable dysfunction and tissue damage after irradiation. DFO restored SFR in the irradiated glands to a level comparable to that in normal glands and angiogenesis in damaged tissue was greatly increased. DFO also increased the expression levels of HIF-1α and VEGF while reduced apoptotic cells. Furthermore, Sca-1^+^cells were preserved in the salivary glands treated with DFO before IR.

**Conclusions:**

Our results indicate DFO could prevent the radiation-induced dysfunction of salivary glands in mice. The mechanism of this protective effect may involve increased angiogenesis, reduced apoptosis of acinar cells and more preserved stem cells.

## Introduction

Oral and maxillofacial malignant tumors, which occur in the lip, oral cavity, paranasal sinuses and salivary glands, account for 644,000 patients of all new cancer cases each year in the world. A majority of patients are treated with radiotherapy, which is considered one of the most effective treatments, either alone or in combination with other treatments such as surgery and/or chemotherapy [1]. Because of its special anatomic location and sensitivity to irradiation, the salivary glands (SG) are always injured during irradiation (IR) therapy. Progressive loss of function may occur within the first weeks of radiotherapy and can persist for life [2]. Radiation-induced salivary gland dysfunction may cause dental caries, difficulties in speaking and swallowing, mucositis, and xerostomia (dry mouth syndrome), which may severely compromise the life quality of these patients [3], [4].

The underlying mechanism of the IR-induced injury to SGs remains unclear. The possibility that microvascular endothelial cells might be targeted by IR was first revealed in gastrointestinal cancer by Paris et al. [5]. Later, several following studies found that endothelium of blood vessel was also damaged by radiation during treatment of lung and brain cancers [6], [7]. More importantly, Cotrim et al showed that reduction of microvessel density in murine SGs occurred 4 hours after IR, indicating the injury of endothelial cells. To overcome the drawback of irradiation therapy, transfer of vascular endothelial growth factor (VEGF) and basic fibroblast growth factor (bFGF) complementary DNAs to endothelial cells by means of vectors was carried out to enhance angiogenesis in damaged tissue. It was found secretion of salivary fluid by SG was greatly restored with enhanced capillary density and more survived endothelial cells after irradiation [8]. However, gene transfer therapy is complicated and brings great safety concerns in clinical application. Thus, to explore an alternative approach to protect SG from irradiation injury is very important for treating oral and maxillofacial malignant tumors.

Deferoxamine (DFO), a bacteria-derived siderophore from actinobacter Streptomyces pilosus, has been used in the treatment of the diseases with excess iron, such as hemochromatosis, thalassemia, myeloid dysplasia syndrome and chronic iron overload, as well as in treating the patients suffering from an overload of aluminum during a continuous kidney dialysis. In addition to the iron-chelating function, DFO administration was associated with up-regulated expression of vascular endothelial growth factor (VEGF) in both normal tissues and malignant tumors [9]. Further studies revealed that application of DFO could accelerate angiogenesis in ischemic tissue via VEGF-mediated pathway [10], [11]. However, whether DFO administration would improve angiogenesis, thus protecting salivary gland from irradiation damage remains unclear.

Here, the aim of the present study is to evaluate the effects of DFO administration on the salivary gland function after irradiation in mouse model. Meanwhile, the potential mechanism of the functional improvement of irradiation-damaged salivary glands was investigated.

## Materials and Methods

### Irradiation of Salivary Glands

A Total of 55 male C_57_BL/6 mice (8–12 week old and 20–25 g in body weight) were purchased from SLAC National Rodent Laboratory Animal Resources (Shanghai, China). The mice were kept under clean conventional conditions and fed with food pellets and acidified tap water (pH = 2.8). This study was carried out in strict accordance with the recommendations of the Guide for the Care and Use of Laboratory Animals of the National Institutes of Health. The protocol was approved by the Committee on the Ethics of Animal Experiments of the Ninth People’s Hospital, affiliated to Shanghai Jiao Tong University School of Medicine, China. The mice were anesthetized by intraperitoneal injection of 0.4 ml/100 g chloral hydrate. Submandibular gland was then subjected to point-fixed irradiation with a single dose of 18 Gy by photon MLC linear accelerator (Elekta, Swedish). The radiation dose is known to induce sufficient reduction of salivary flow rate without affecting general health of the animals [12].

### Administration of DFO

**Figure 1:**
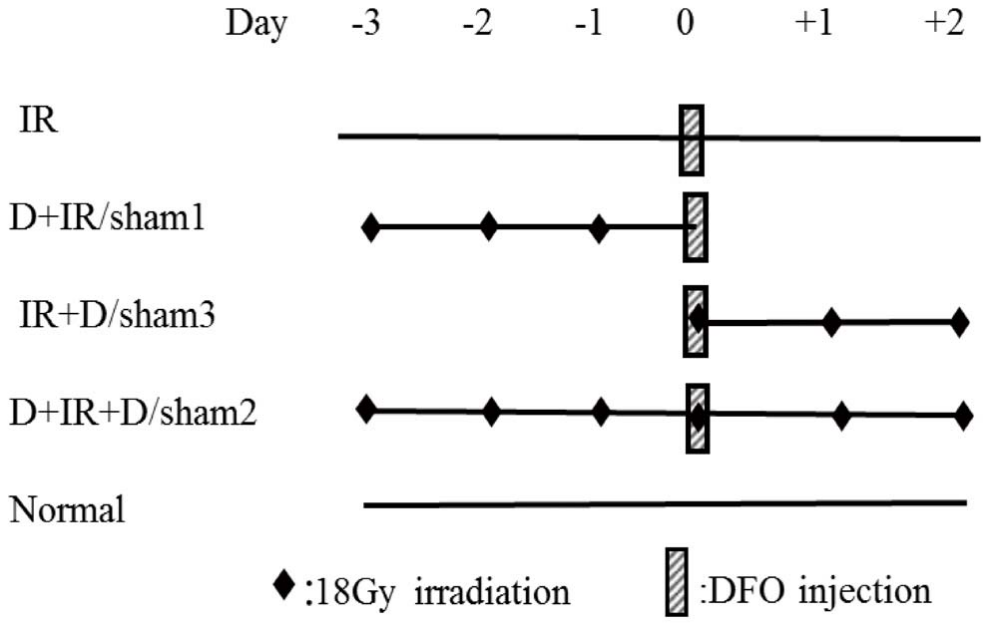
Schematic representation of the experimental design. Local 18 Gy irradiation of salivary glands was given before or/and after 3-day DFO treatment (50 mg/kg/d). Normal: no treatment; IR: only Irradiation group; D+IR: Pre-DFO group; sham1: Pre-sterilized water group; IR+D: Post-DFO group; sham3: Post-sterilized water group; D+IR+D: Pre+Post DFO group; sham2: Pre+Post sterilized water group; DFO: deferoxamine.

The mice were divided into several groups: (1) Normal group: the mice that received no treatment (n = 5); (2) Irradiation group (IR): the mice that received a single dose of 18 Gy of irradiation (n = 5); (3) Pre-DFO group (D+IR): the mice that received daily injection of DFO for 3 days before irradiation (n = 10); (4) Pre+Post DFO group (D+IR+D): the mice that received daily injection of DFO for 3 days both before and after irradiation (n = 10); (5) Post-DFO group (IR+D): the mice that received daily injection of DFO for 3 days after irradiation (n = 10). (6) For each DFO-treated group, their sham control mice were intraperitoneally injected with 0.1ml sterilized water (vehicle control) for 3 days before or/and after irradiation and served as sham control. Sham1: Pre-sterilized water group (n = 5); sham2: Pre+Post sterilized water group (n = 5); sham3: Post-sterilized water group (n = 5) (Figure 1). DFO administration was performed as its indication and usage. Briefly, 500 mg of desferrioxamine mesylate (Beijing Novartis Pharma Ltd) was dissolved in 40 ml of sterilized water and was then intraperitoneally injected daily at a dose of 50 mg/kg. We chose a safety dosage of 50 mg/kg/d based on the fact that intravenous LD50 of desferrioxamine mesylate in mice is 287 mg/kg as shown in the instruction manual [13].

### Secretory function evaluation of salivary glands

The mice were injected with pilocarpine chloride (2 mg/kg i.p) under anesthesia to stimulate secretion of saliva, at 30^th^, 60^th^, and 90^th^ day after irradiation, respectively. Two minutes after injection, the whole volume of saliva was collected by five small pre-weighed cotton balls that were placed under the tongue for 10 minutes. Immediately after soaked with saliva, the cotton balls were removed from the mouth and weighed. The volume of saliva was determined gravimetrically, assuming a density of 1 g/ml for saliva [14].

### Histological observation

Ninety days after irradiation, the mice were sacrificed by cervical dislocation and their submandibular glands were harvested and immediately weighed, followed by fixation in 4% paraformaldehyde, dehydrated and embedded in paraffin. Structures of the glands were analyzed by H&E staining. The functionalities of acinar cells were determined using periodic acid-Schiff’s base staining (PAS, Sigma-Aldrich, USA; 395B). The surface area of PAS-positive acinar cells was examined from two different sections (the upper and the middle) of each submandibular gland and at least 3 fields in each section were assessed blindly by two independent investigators. The software Image-Pro Plus 6.0 was used to analyze the surface area occupied by acinar cells.

### Immnunohistochemical staining

Tissue sections with 5 um thickness were deparaffinized and rehydrated. After treated with 3% of H_2_O_2_ for 15 minutes at the room temperature to block endogenous peroxidase, the sections were blocked in 0.1% trypsin and 1% bovine serum albumin (BSA, Sigma-Aldrich, USA) for 30 minutes at 37°, and then incubated with the primary antibodies against CD_31_ (Abcam, UK; ab28364), Sca-1 (Abcam, UK; ab51317) or PCNA (CST, USA; 2586) at 4°C overnight. After rinsed with PBS, sections were incubated with the second antibody (goat anti-mouse IgG, Roche, USA) for 30 minutes at 37°C and subsequently developed with Liquid DAB Substrate Chromogen System (Roche, USA) for 5 minutes. The nucleus was then counterstained with hematoxylin for 2 minutes. For quantification of blood vessel density, the percentage of surface area occupied by blood vessels indicated by CD_31_ positive staining was assessed under 100× magnification chosen from the two different sections (the upper and the middle) of each submandibular gland. Blood vessel densities were calculated using Image-Pro Plus 6.0 software from at least 3 fields in each section.

For detection of cell proliferation, PCNA positive cells within 3 representative fields from each section were counted independently. Two sections were chosen from each gland examined.

### Cellular Apoptosis detected by TUNEL

Cell apoptosis was assessed by the terminal deoxynucleotide transferase mediated ddUTP nick end labeling (TUNEL, Roche, USA; 11684817910) assay according to manufacturer’s instruction. Briefly, apoptotic nuclear DNA strand breaks were end labeled with digoxigenin conjugated dideoxy-UTP by terminal transferase and visualized immunohistochemically with digoxigenin antibody conjugated to alkaline phosphatase. The assay was standardized with the use of adjacent tissue sections treated with DNase I to induce DNA fragmentation as a positive control for apoptosis. Apoptotic cells were identified by the presence of apoptotic bodies in the cell. The TUNEL-positive cells were assessed under 200× magnification chosen from the two different sections (the upper and the middle) of each submandibular gland. Ratio of TUNEL-positive cells to total nuclei (% per gland) were calculated using Image-Pro Plus 6.0 software from at least 3 fields in each section.

### Quantitative Real-Time (PCR)

Total RNA was extracted with Trizol from submandibular glands harvested at indicated time points. Three micrograms of RNA were reversely transcribed into cDNA using AMV Reverse transcriptase (PROMEGA, USA; M5101) and Real-time PCR kit (TAKARA, Japan; DRR420A). Real-time PCR was performed at 57°C for 30 cycles in the Opticon Continuous Fluorescent Detector using StepOnePlus PCR system (Applied BioSystems, USA). Triplicates were performed for each sample, and results were normalized to β-actin level. The following primers were used. β-actin: F5′ACC CCCACTGATACGCCTGA3′, R5′ TGAGCACTGAAGCGAAAGC3′. VEGF: F5′ACGAAGCGCAAGAAATCCC3′, R5′TTAACTCAAGCTGCCTCGCC3′.

### Western Blot analysis

Western blot was performed to evaluate HIF-1α accumulation in gland tissue. Nuclear extracts (100 ug) were electrophoresed through 5% sodium dodecyl sulfate (SDS)-polyacrylamide gels and electrotransferred onto PVDF membrane. Blots were incubated with primary antibody against HIF-1α (Abcam, UK; ab8366) and β-actin (CST, USA; 4970). Secondary antibody of goat anti-mouse IgG (CST, USA; 7076) was applied and visualized using chemiluminescence (HRP), and then exposed to X-ray films. Protein expression levels were quantified by densitometry.

### Statistical Analysis

Data were expressed as mean±standard error of mean (mean±SEM). For comparison among different groups, statistical significance was assessed using a one-way analysis of variance (ANOVA) and the significance of each difference was determined by post hoc testing using LSD. Statistical significance was considered at P<0.05. Statistical analysis was performed with SPSS16.0.

## Result

### DFO attenuated Radiation-induced Hyposalivation

**Figure 2:**
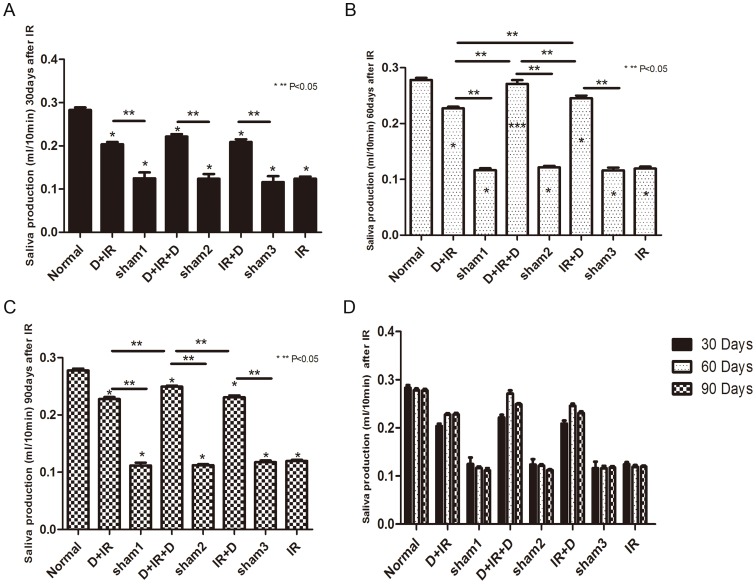
Salivary flow rate (SFR) was calculated at 30^th^, 60^th^, and 90^th^ day after irradiation. Sham1: Pre-sterilized water group; sham2: Pre+Post sterilized water group; sham3: Post-sterilized water group. Data is presented as means ± SEM. *: P<0.05 compared with normal group; **: P<0.05 between two individual groups; ***: P>0.05 compared with normal group.

**Figure 3:**
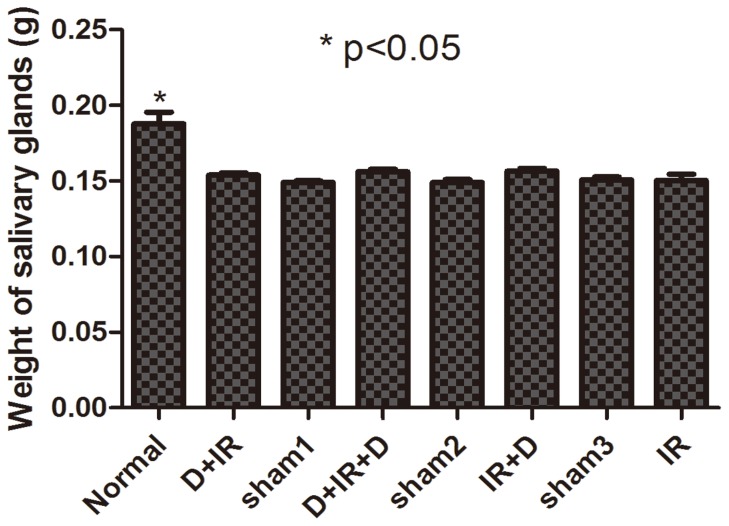
The weight of salivary gland harvested at 90^th^ day post irradiation. The weight of salivary gland shows no significant difference among individual groups. Sham1: Pre-sterilized water group; sham2: Pre+Post sterilized water group; sham3: Post-sterilized water group. Data is presented as means ± SEM. *: P<0.05 compared with normal group.

To investigate whether DFO administration could attenuate hyposalivation resulted from radiation injury, salivary flow rate (SFR) was calculated at 30^th^, 60^th^, and 90^th^ day after the point-fixed irradiation at a single dose of 18 Gy, respectively (The original data were shown in Table S1). Irradiated mice without DFO administration developed severe hyposalivation. In contrast, DFO treatment, irrespective of the treatment schedule, resulted in significantly higher SFR in irradiated mice than vehicle-treated animals (P<0.05; Fig. 2D). Moreover, we found that, at 30^th^ day after irradiation, each group that had received DFO treatment showed similar SFR (P>0.05; Fig. 2A). Sixty days after irradiation, the D+IR+D group preserved the highest SFR (97%) which was comparable to those of normal non-irradiated control mice (P>0.05; Fig. 2B). Although there was a slight reduction at 90^th^ day after irradiation, SRF of D+IR+D group maintained the highest level among all DFO-treated groups (P<0.05; Fig. 2C). However, 90 days after irradiation, the weight of salivary glands harvested showed no significant difference between individual groups (P>0.05; Fig. 3. The original data were shown in Table S2).

### Anti-apoptosis effect of DFO in irradiation damaged tissue

**Figure 4:**
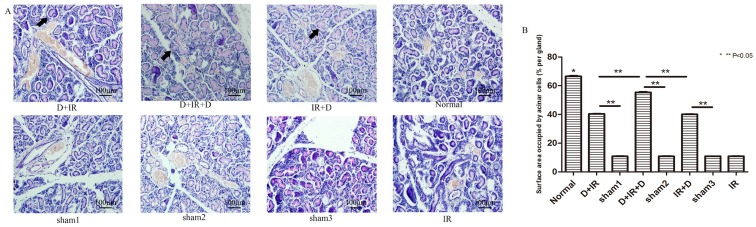
PAS staining and Surface area occupied by acinar cells determination at 90^th^ day after irradiation. A: PAS staining of submandibular gland tissue from each group. Arrows indicate functional acinar cells. B: Surface area occupied by acinar cells (% per gland) between different groups. Sham1: Pre-sterilized water group; sham2: Pre+Post sterilized water group; sham3: Post-sterilized water group. Data is presented as means ± SEM. *: P<0.05 compared with normal group; **: P<0.05 between two individual groups.

Ninety days after irradiation, massive acinar cells were clearly delepted within gland tissue in all irradiation-treated groups that received no DFO administration, indicating delayed and continuous irradiation damage in gland tissue. In contrast, DFO administration reduced the loss of acinar cells in gland tissue. The surface area of functional acinar cells increased to 61.05%, 83.17% and 60.22% compared with that of normal SGs in D+IR, D+IR+D and IR+D respectively, among which surface area in D+IR+D group showed the greatest increase (P<0.05; Fig. 4. The original data were shown in Table S3).

**Figure 5:**
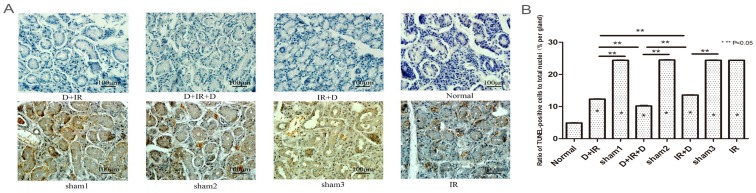
Analysis of apoptotic cells by TUNEL determination at 90^th^ day after irradiation. TUNEL assay of apoptotic cells in different groups. A: TUNEL-positive cells of submandibular gland tissue from each group. B: Ratio of TUNEL-positive cells to total nuclei (% per gland) between different groups. Sham1: Pre-sterilized water group; sham2: Pre+Post sterilized water group; sham3: Post-sterilized water group. Data is presented as means ± SEM. *: P<0.05 compared with normal group; **: P<0.05 between two individual groups.

**Figure 6:**
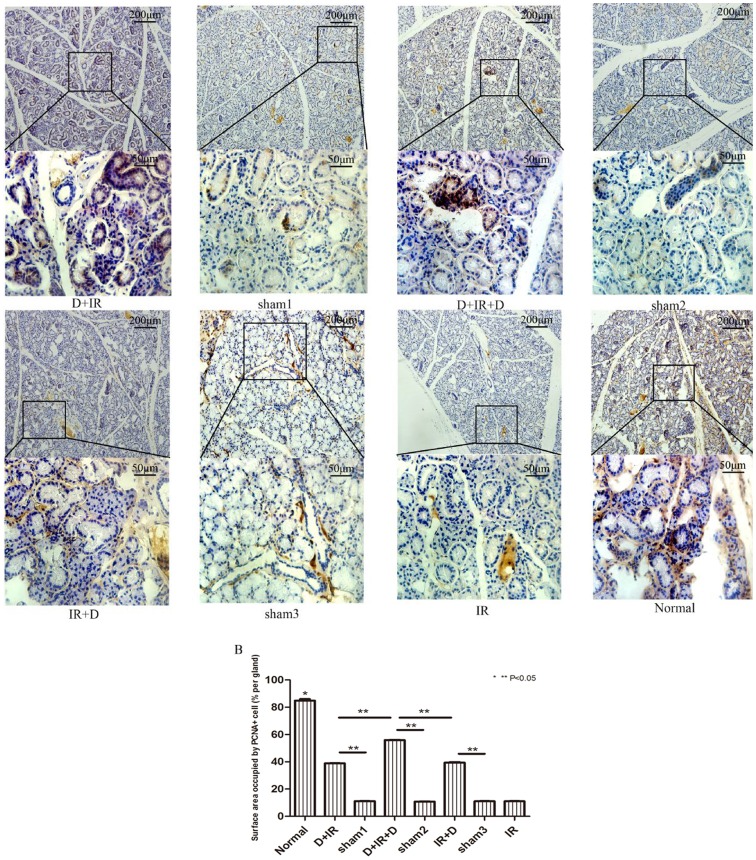
Detection of proliferating cells in submandibular gland tissue at 90^th^ day after irradiation. A: Immunohistochemical staining of PCNA from each group. Images of lower panel represent the higher magnification of the boxed area in corresponding images of upper panel. B: Surface area occupied by PCNA-positive cells (% per gland) between different groups. Sham1: Pre-sterilized water group; sham2: Pre+Post sterilized water group; sham3: Post-sterilized water group. Data is presented as means ± SEM. *: P<0.05 compared with normal group; **: P<0.05 between two individual groups.

TUNEL analysis was carried out to determine the effect of DFO in the prevention of cell apoptosis in irradiated salivary glands. Ninety days after irradiation, TUNEL assay revealed that apoptotic cells in irradiated salivary glands increased significantly compared to those in normal SGs (P<0.05); The TUNEL positive cells was remarkably reduced in each DFO-treated group as compared to their vehicle groups (P<0.05; Fig. 5. The original data were shown in Table S4). Moreover, as determined by PCNA staining, the number of proliferating cells was significantly increased in DFO-treated groups (P<0.05; Fig. 6. The original data were shown in Table S5). Again, the lowest number of apoptotic cells and highest number of proliferating cells were observed in D+IR+D group (Fig. 5 & Fig. 6).

### DFO improved angiogenesis via activating HIF-1α-VEGF Pathway

**Figure 7:**
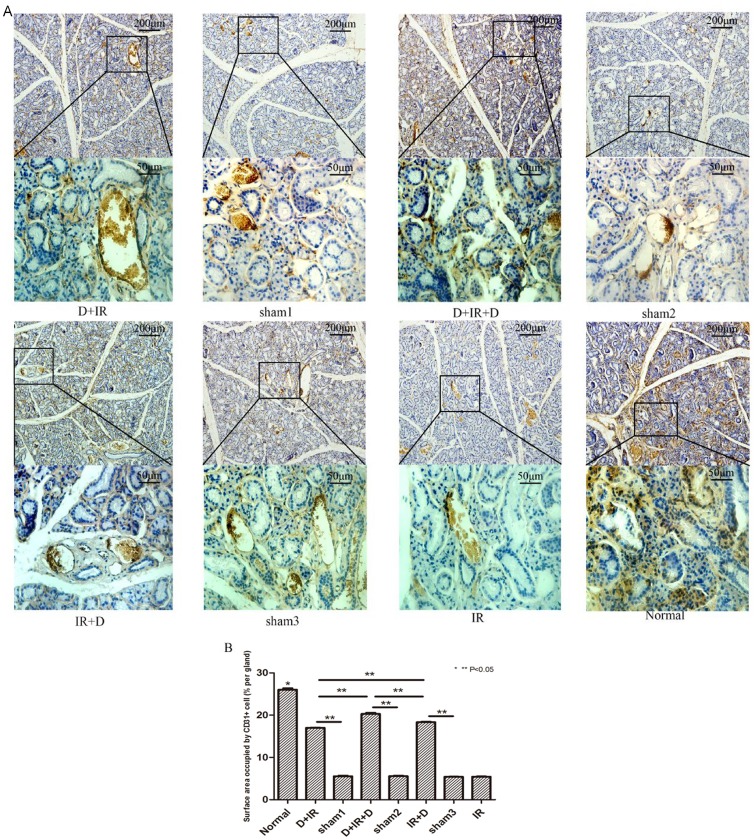
Effect of DFO administration on angiogenesis in irradiated submandibular gland. A: Immunohistochemical staining of CD_31_ in different groups. Images of lower panel represent the higher magnification of the boxed area in corresponding images of upper panel. B: Surface area occupied by CD_31_^+^ cells (% per gland) between different groups. Sham1: Pre-sterilized water group; sham2: Pre+Post sterilized water group; sham3: Post-sterilized water group. Data is presented as means ± SEM. *: P<0.05 compared with normal group; **: P<0.05 between two individual groups.

To investigate whether the protective effect of DFO on SG function was accompanied with enhanced angiogenesis, we performed calculation of capillary density and surface area occupied by CD_31_ positive cells in salivary glands. H&E staining (Data not shown) showed that density of capillary containing erythrocytes was much higher in DFO-treated groups than that in vehicle-treated groups. By more specific immunohistochemistry for CD_31_, it was found that the surface area of CD_31_ positive cells was much larger in DFO treated groups, among which surface area in D+IR+D group was highest, reaching 77.93% of that in normal group (P<0.05; Fig. 7. The original data were shown in Table S6).

**Figure 8:**
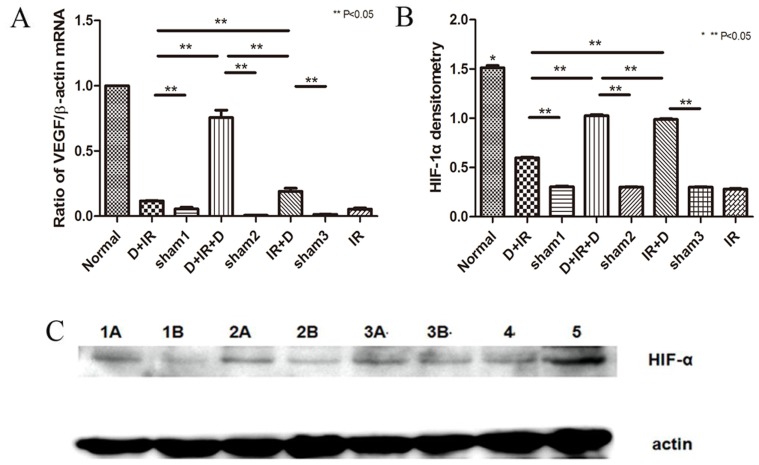
DFO administration improved angiogenesis in irradiated tissue via activating HIF-1α-VEGF Pathway. A: Real-time PCR detection of VEGF expression 90 days after irradiation. B: Western-blot analysis of HIF-1α in tissues received DFO. Sham1: Pre-sterilized water group; sham2: Pre+Post sterilized water group; sham3: Post-sterilized water group. Data is presented as means ± SEM. *: P<0.05 compared with normal group; **: P<0.05 between two individual groups. C: HIF-1α in each group. Annotate: 1A represents D+IR group; 1B represents sham1; 2A represents IR+D group; 2B represents sham3; 3A represents D+IR+D group; 3B represents sham2; 4 represents IR; 5 represents normal group; β-Actin represents reference.

To evaluate whether increased vascular density resulted from elevated expression of VEGF, we examined VEGF expression in the salivary glands after DFO treatment. We found that VEGF mRNA level was increased in all DFO treated groups, among which D+IR+D group showed the highest level (P<0.05; Fig. 8A. The original data were shown in Table S7). We also performed western blot to assess the protein level of HIF-1α, the master regulator of VEGF expression, in salivary gland. Similarly, the highest expression of HIF-1α was found in D+IR+D group (P<0.05; Fig. 8B, C. The original data were shown in Table S7).

### DFO improved survival of salivary stem/progenitor cells

**Figure 9:**
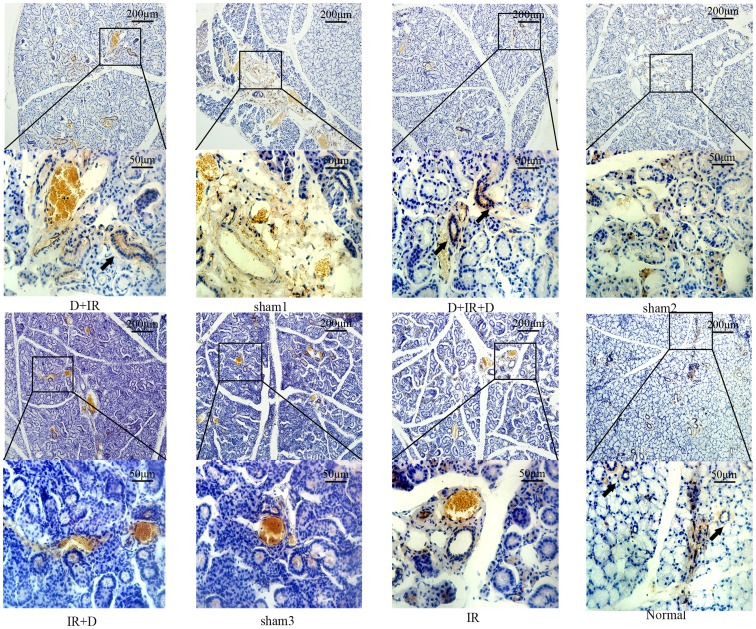
DFO administration improved survival of salivary stem/progenitor cells. Immunohistochemical staining of Sca-1 in different groups. Images of lower panel represent the higher magnification of the boxed area in corresponding images of upper panel. Arrows indicate Sca-1^+^ cells.

To determine whether DFO administration could promote the survival of salivary stem cells, we detected Sca-1 positive cells, which had been widely accepted as stem/progenitor cells in SG [15], by immunohistochemistry staining at 90^th^ day post irradiation. Sca-1^+^ cells could be easily detected in normal salivary glands and the salivary glands from groups that received DFO administration before irradiation (D+IR and D+IR+D groups). Meanwhile, few Sca-1^+^cells could be detected in the group treated with irradiation alone or in the group that received DFO administration after irradiation (IR and IR+D group) (Fig. 9).

## Discussion

In the present study, we have demonstrated the efficacy of DFO for correcting the dysfunction of salivary glands induced by point-fixed irradiation. We found that, in addition to the restoration of salivary function, DFO administration also increased angiogenesis and the number of stem/progenitor cells while reduced the apoptosis of acinar cells.

In our study, SFR in all DFO-treated groups was higher than that in vehicle-treated groups, demonstrating the therapeutic effect of DFO in restoring the function of irradiated glands. SFR is a common parameter for evaluating secretion function of salivary gland due to it is a noninvasive, simple approach with minimal infection risk that can reduce the mortality of mice. However, SFR appears to be influenced by the intake of food and water of irradiated animals during the experimental period [16]. Moreover, the collected saliva represented the sum of the saliva from both parotid and submandibular glands. In the current study, a point-fixed irradiation (18 Gy) was given only to the submandibular glands and the parotid received little irradiation. Thus, the reduction of salivary flow is mainly resulted from the irradiation damage to the submandibular glands. It’s worth noting that (1) there is a much higher prevalence of serous cells in the parotid gland as compared with the submandibular gland, the parotid gland exhibits higher radiosensitivity, while salivary gland is relative radioresistant; (2) In a 1 year follow-up study, it was observed that irradiation-induced salivary hypofunction was more severe in parotid gland than in submandibular gland at each time point examined; (3) although most of the reduction in salivary function is achieved within 3 months following irradiation, the parotid gland showed a substantial dysfunction during the first 2 weeks, which was not observed in submandibular gland [17]. Collectively, these data implicate the different response of parotid and submandibular glands to irradiation and more detailed investigation are needed to explore the potential therapeutic effect of DFO on the two glands, respectively.

In addition, we found that the highest SFR recovery induced by DFO administration occurred 60 days after irradiation and it was slightly decreased after 90 days. In agreement with our study, Lim et al recently reported a decline of SRF 12 weeks (84 days) after irradiation in a similar irradiated mice model, which was corrected by systemically administration of adipose derived stem cells immediately after the irradiation [18]. Thus, our results indicate that the therapeutic effect of DFO occurred within the duration of 60–90 days after irradiation. Beyond this period of time, and with no further DFO administration conducted, the initial therapeutic effect may gradually decay. Thereby, to investigate whether treatment effects are short term or continuous, and to stabilize its effects on salivary flow for a long-term therapy, it will be helpful to extend the observation time to 120 or 150 days in future studies to find a more appropriate and effective time-interval for DFO administration.

By calculating the capillary density, detecting the expression of VEGF and HIF-1α, we found that DFO may stimulate angiogenesis via increasing HIF-1α level, which consequently leads to the up-regulation of VEGF expression. Furthermore, the improved function of irradiated glands by DFO administration was in parallel to the enhanced angiogenesis in the damaged tissue. Consistent with these results, Kojima et al observed that improved salivary flow rates occurred with increased expression of VEGF by injection of adipose-derived stem cells into irradiation damaged glands [19]. However, Nagler et al. found that long-term (2 months after irradiation) parotid function was partially protected against irradiation by the pre-irradiation administration of zinc-desferoxamine (Zn-DFO) at a dose of 20 mg/kg. While the submandibular glands, in contrast to the parotids, were not protected by Zn-DFO. They attributed this protective effect of Zn-DFO to chelation of the intracellular redox-active metal ions [20]. We speculated the difference in animal models (rat versus mice), doses of DFO (20 mg/kg for 1 time versus 50 mg/kg for 3 times), and timing of injection (pre-irradiation versus pre- and post-irradiation) may cause the discrepancies between Nagler’s and our studies. Also, the group size of our animal study is relatively small. We found the mortality rate of the irradiated mice without DFO administration was high. Thus, we have to make a balance between the optimal sample size and the ethics of animal experiment and finally we chose 5 animals in control groups. The irradiated mice with DFO injection showed slightly high survival rate, so we chose 10 as the sample size of the DFO-treated groups.

In the present study, we also observed some Sca-1^+^ salivary stem cells were preserved only in D+IR and D+IR+D groups. We, thus, suppose DFO administration before radiotherapy could promote survival of salivary stem cells in the irradiated-SGs. The stem cells which had suffered from irreversible damage of irradiation would not survive, even though DFO was administrated after radiotherapy. The effect of DFO on maintenance of stem cells through suppression of proliferation has been documented in a study of cardiac stem cell by Song et al [21]. Thus, in the present study, we present the in-vivo evidence supporting the in-vitro effects of DFO on stem cells.

Quite a few experimental studies as well as clinical trials have demonstrated that some aggressive tumors [22]–[24], particularly neuroblastoma [25] and leukemia [26], are sensitive to Fe chelation therapy such as DFO. Additionally, DFO administration has been proved to be able to inhibit the growth and/or induce the apoptosis of malignant cells from leukaemia [27], [28], neuroblastoma [29], [30], melanoma [31], hepatoma [32], Kaposi’s sarcoma [33] and cervical cancer [34]. More importantly, it was found that DFO could inhibit the growth of oral squamous carcinoma cell lines [35]. Given all these findings, we propose that DFO administration would protect SGs from irradiation damage with additional benefit in inhibiting the growth of head and neck tumor. However, there are distinct differences in structure and physiological functions of salivary glands between human and mouse. For example, saliva is secreted only following stimulation in rodents, while in humans saliva is secreted spontaneously without stimulation. Furthermore, serous cell death resulted from irradiation in human salivary glands is predominant and accompanied with infiltration of inflammatory cells, which could not be observed in rats [17]. Thus, the potential benefits of DFO to the treatment of head and neck tumor remains to be tested in human.

In conclusion, our study provided the evidence that DFO administration could protect salivary glands from radiation damage in a mice model. The improvement in function of salivary glands is accompanied with reduced apoptotic acinar cells, enhanced angiogenesis and more survived Sca-1^+^ salivary stem cells. The potential of our finding as an alternative therapeutic approach to prevent radiation-induced dysfunction of salivary glands in clinical application deserves more investigation in future.

## Supporting Information

Table S1 Salivary flow rate (SFR) of each mouse was calculated at 30^th^, 60^th^, and 90^th^ day after the point-fixed irradiation with a single dose of 18 Gy. Sham1: Pre-sterilized water group; sham2: Pre+Post sterilized water group; sham3: Post-sterilized water group.
(DOC)

Table S2 The weight of each salivary gland harvested at 90^th^ day post irradiation. Sham1: Pre-sterilized water group; sham2: Pre+Post sterilized water group; sham3: Post-sterilized water group.
(DOC)

Table S3 Surface area occupied by acinar cells (% per gland) of each salivary gland. Sham1: Pre-sterilized water group; sham2: Pre+Post sterilized water group; sham3: Post-sterilized water group. The software Image-Pro Plus 6.0 was used to analyze the surface area occupied by acinar cells.
(DOC)

Table S4 Ratio of TUNEL-positive cells to total nuclei (% per gland) of each salivary gland. Sham1:Pre-sterilized water group; sham2: Pre+Post sterilized water group; sham3: Post-sterilized water group. The software Image-Pro Plus 6.0 was used to analyze the ratio of TUNEL-positive cells to total nuclei.
(DOC)

Table S5 Surface area occupied by PCNA-positive cells (% per gland) of each salivary gland. Sham1: Pre-sterilized water group; sham2: Pre+Post sterilized water group; sham3: Post-sterilized water group. The software Image-Pro Plus 6.0 was used to analyze the surface area occupied by PCNA^+^ cells.
(DOC)

Table S6 Surface area occupied by CD31^+^ cells (% per gland) of each salivary gland. Sham1: Pre-sterilized water group; sham2: Pre+Post sterilized water group; sham3: Post-sterilized water group. The software Image-Pro Plus 6.0 was used to analyze the surface area occupied by CD31^+^ cells.
(DOC)

Table S7 Real-time PCR detection of VEGF expression and Western-blot analysis of HIF-1α 90 days after irradiation. We showed the value of ΔCT of VEGF and HIF-1α densitometry in each group. Sham1: Pre-sterilized water group; sham2: Pre+Post sterilized water group; sham3: Post-sterilized water group.
(DOC)

## References

[1] The protocol of treatment guideline for oral and maxillofacial malignant neoplasms (2010) Division of Oral and Maxillofacial Oncology. . Chinese Society of Oral and Maxillofacial Surgery. 03:395–403.

[2] Burlage FR, Coppes RP, Meertens H, Stokman MA, Vissink A (2001) Parotid and submandibular/sublingual salivary flow during high dose radiotherapy. . Radiother Oncol. 61:271–274. 11730996 10.1016/s0167-8140(01)00427-3

[3] Vissink A, Jansma J, Spijkervet FK, Burlage FR, Coppes RP (2003) Oral sequelae of head and neck radiotherapy. . Critical Reviews in Oral Biology and Medicine. 14:199–212. 12799323 10.1177/154411130301400305

[4] Vissink A, Burlage FR, Spijkervet FK, Jansma J, Coppes RP (2003) Prevention and treatment of the consequences of head and neck radiotherapy. . Critical Reviews in Oral Biology and Medicine. 14:213–225. 12799324 10.1177/154411130301400306

[5] Paris F, Fuks Z, Kang A, Capodieci P, Juan G, et al (2001) Endothelial apoptosis as the primary lesion initiating intestinal radiation damage in mice. . Science. 293:293–297. 11452123 10.1126/science.1060191

[6] Santana P, Pena LA, Haimovitz-Friedman A, Martin S, Green D, et al (1996) Acid sphingomyelinase-deficient human lymphoblasts and mice are defective in radiation-induced apoptosis. . Cell. 86:189–199. 8706124 10.1016/s0092-8674(00)80091-4

[7] Pena LA, Fuks Z, Kolesnick RN (2000) Radiation-induced apoptosis of endothelial cells in the murine central nervous system: protection by fibroblast growth factor and sphingomyelinase deficiency. . Cancer Research. 60:321–327. 10667583

[8] Ana P Cotrim, Anastasia Sowers, James B Mitchell, Bruce J Baum (2007) Prevention of Irradiation-induced Salivary Hypofunction by Microvessel Protection in Mouse Salivary Glands. . Molecular Therapy. 15:2101–2106. 17726456 10.1038/sj.mt.6300296

[9] Beerepoot LV, Shima DT, Kuroki M, Yeo KT, Voest EE (1996) Up-regulation of vascular endothelial growth factor production by iron chelators. . Cancer Research. 56:3747–3751. 8706019

[10] Ikeda Y, Tajima S, Yoshida S, Yamano N, Kihira Y, et al (2011) Deferoxamine promotes angiogenesis via the activation of vascular endothelial cell function. . Atherosclerosis. 215:339–347. 21315355 10.1016/j.atherosclerosis.2011.01.009

[11] Shen X, Wan C, Ramaswamy G, Mavalli M, Wang Y, et al (2009) Prolyl hydroxylase inhibitors increase neoangiogenesis and callus formation following femur fracture in mice. . Journal of Orthopaedic Research. 27:1298–1305. 19338032 10.1002/jor.20886 PMC3767389

[12] Sumita Y, Liu Y, Khalili S, Maria OM, Xia D, et al (2011) Bone marrow-derived cells rescue salivary gland function in mice with head and neck irradiation. The International Journal of Biochemistry&Cell Biology. 43:80–87. 20933096 10.1016/j.biocel.2010.09.023 PMC3403826

[13] Desferal Prescribing Information. Rxlist website. Available: http://www.rxlist.com/desferal-drug/overdosage-contraindications.htm. Accessed 2014 Oct.1st.

[14] Oki T, Takeuchi C, Yamada S (2006) Comparative evaluation of exocrine muscarinic receptor binding characteristics and inhibition of salivation of solifenacin in mice. . Biological& Pharmaceutical Bulletin. 29:1397–1400. 16819176 10.1248/bpb.29.1397

[15] Lombaert IM, Brunsting JF, Wierenga PK, Faber H, Stokman MA, et al (2008) Rescue of salivary gland function after stem cell transplantation in irradiated glands. PLoS ONE 3:e2063 10.1371/journal.pone.0002063 18446241 PMC2329592

[16] Nagler RM, Baum BJ, Fox PC (1993) Acute effects of X irradiation on the function of rat salivary glands. . Journal of Radiation Research. 136:42–7. 8210337

[17] Nagler RM (2002) The enigmatic mechanism of irradiation-induced damage to the major salivary glands. . Oral Diseases. 8:141–146. 12108758 10.1034/j.1601-0825.2002.02838.x

[18] Lim JY, Ra JC, Shin IS, Jang YH, An HY, et al (2013) Systemic transplantation of human adipose tissue-derived mesenchymal stem cells for the regeneration of irradiation-induced salivary gland damage. . PLoS One. 8:e71167 10.1371/journal.pone.0071167 23951100 PMC3739795

[19] Kojima T, Kanemaru S, Hirano S, Tateya I, Ohno S, et al (2011) Regeneration of radiation damaged salivary glands with adipose-derived stromal cells. . Laryngoscope. 121:1864–1869. 21748735 10.1002/lary.22080

[20] Nagler RM, Marmary Y, Golan E, Chevion M (1998) Novel protection strategy against X-ray-induced damage to salivary glands. . Journal of Radiation Research. 149:271–6. 9496890

[21] Song D, Li Y, Cao J, Han Z, Gao L, et al (2013) Effect of iron deficiency on c-kit^+^ cardiac stem cells in vitro. . PLoS One. 8:e65721 10.1371/journal.pone.0065721 23762416 PMC3677875

[22] Buss JL, Torti FM, Torti SV (2003) The role of iron chelation in cancer therapy. . Current Medicinal Chemistry. 10:1021–1034. 12678674 10.2174/0929867033457638

[23] Richardson DR (2005) Molecular mechanisms of iron uptake by cells and the use of iron chelators for the treatment of cancer. . Current Medicinal Chemistry. 12:2711–2729. 16305467 10.2174/092986705774462996

[24] Pah PM, Horwitz LD (2005) Cell permeable iron chelators as potential cancer chemotherapeutic agents. . Cancer Investigation. 23:683–691. 16377587 10.1080/07357900500359976

[25] Donfrancesco A, Deb G, Dominici C, Pileggi D, Castello MA, Helson L (1990) Effects of a single course of deferoxamine in neuroblastoma patients. . Cancer Research. 50:4929–4930. 2379156

[26] Estrov Z, Tawa A, Wang XH, Dubé ID, Sulh H, et al (1987) In vitro and in vivo effects of deferoxamine in neonatal acute leukemia. . Blood. 69:757–761. 3493042

[27] Haq RU, Werely JP, Chitambar CR (1995) Induction of apoptosis by iron deprivation in human leukemic CCRF-CEM cells. . Experimental Hematology. 23:428–432. 7720813

[28] Fukuchi K, Tomoyasu S, Tsuruoka N, Gomi K (1994) Iron deprivation induced apoptosis in HL-60 cells. . FEBS Letters. 350:139–142. 8062913 10.1016/0014-5793(94)00755-1

[29] Becton DL, Bryles P (1988) Deferoxamine inhibition of human neuroblastoma viability and proliferation. Cancer Research 8:7189–7192. 3191493

[30] Brodie C, Siriwardana G, Lucas J, Schleicher R, Terada N, et al (1993) Neuroblastoma sensitivity to growth inhibition by deferrioxamine:evidence for a block in G1 phase of the cell cycle. . Cancer Research. 53:3968–3975. 8358725

[31] Richardson D, Ponka P, Baker E (1994) The effect of the iron(III) chelator, desferrioxamine, on iron and tranferrin uptake by the human malignant melanoma cell. . Cancer Research. 54:685–689. 8306330

[32] Hann HW, Stahlhut MW, Hann CL (1990) Effect of iron and desferoxamine on cell growth and in vitro ferritin synthesis in human hepatoma cell lines. . Hepatology. 11:566–569. 2158479 10.1002/hep.1840110407

[33] Simonart T, Degraef C, Andrei G, Mosselmans R, Hermans P, et al (2000) Iron chelators inhibit the growth and induce the apoptosis of Kaposi’s sarcoma cells and of their putative endothelial precursors. . The Journal of Investigative Dermatology. 115:893–900. 11069629 10.1046/j.1523-1747.2000.00119.x

[34] Simonart T, Boelaert JR, Mosselmans R, Andrei G, Noel JC, et al (2002) Antiproliferative and apoptotic effects of iron chelators on human cervical carcinoma cells. . Gynecologic Oncology. 85:95–102. 11925126 10.1006/gyno.2001.6570

[35] Lee SK, Jang HJ, Lee HJ, Lee J, Jeon BH, et al (2006) p38 and ERK MAP kinase mediates iron chelator-induced apoptosis and -suppressed differentiation of immortalized and malignant human oral keratinocytes. Life Sciences. 79:1419–1427. 16697418 10.1016/j.lfs.2006.04.011

