# Supplementary material for: Restoring the Secretory Function of Irradiation-Damaged Salivary Gland by Administrating Deferoxamine in Mice
Source: PLoS One. 2014 Nov 26;9(11):e113721. doi: 10.1371/journal.pone.0113721 (PMC4245233; doi:10.1371/journal.pone.0113721)
Supplement: Table S1 — Salivary flow rate (SFR) of each mouse was calculated at 30th, 60th, and 90th day after the point-fixed irradiation with a single dose of 18 Gy. Sham1: Pre-sterilized water group; sham2: Pre+Post sterilized water group; sham3: Post-sterilized water group. (DOC) [file pone.0113721.s001.doc]

**Table S1: Salivary flow rate (SFR) of each mouse was calculated at 30^th^, 60^th^, and 90^th^ day after the point-fixed irradiation with a single dose of 18 Gy.** Sham1: Pre-sterilized water group; sham2: Pre+Post sterilized water group; sham3: Post-sterilized water group.

| Group | SFR 30 days after IR （ml/10min) | SFR 60 days after IR （ml/10min) | SFR 90 days after IR （ml/10min) |
| --- | --- | --- | --- |
| Normal | 0.2743 | 0.2665 | 0.2734 |
| Normal | 0.2757 | 0.2749 | 0.2812 |
| Normal | 0.2976 | 0.2804 | 0.2879 |
| Normal | 0.2966 | 0.2802 | 0.2757 |
| Normal | 0.2709 | 0.2886 | 0.2696 |
| D+IR | 0.1842 | 0.2210 | 0.2261 |
| D+IR | 0.1994 | 0.2258 | 0.2310 |
| D+IR | 0.2171 | 0.2318 | 0.2259 |
| D+IR | 0.2001 | 0.2113 | 0.2371 |
| D+IR | 0.2385 | 0.2281 | 0.2436 |
| D+IR | 0.2039 | 0.2274 | 0.2277 |
| D+IR | 0.2041 | 0.2275 | 0.2276 |
| D+IR | 0.2038 | 0.2273 | 0.2278 |
| D+IR | 0.2040 | 0.2276 | 0.2277 |
| D+IR | 0.1846 | 0.2468 | 0.2025 |
| sham1 | 0.0772 | 0.1094 | 0.0970 |
| sham1 | 0.1203 | 0.1119 | 0.1098 |
| sham1 | 0.1565 | 0.1276 | 0.1284 |
| sham1 | 0.1249 | 0.1167 | 0.1115 |
| sham1 | 0.1459 | 0.1179 | 0.1110 |
| D+ID+D | 0.1946 | 0.2300 | 0.2417 |
| D+ID+D | 0.2092 | 0.3002 | 0.2582 |
| D+ID+D | 0.2475 | 0.2694 | 0.2567 |
| D+ID+D | 0.2234 | 0.2648 | 0.2411 |
| D+ID+D | 0.2523 | 0.2670 | 0.2448 |
| D+ID+D | 0.2175 | 0.2698 | 0.2542 |
| D+ID+D | 0.2110 | 0.2622 | 0.2522 |
| D+ID+D | 0.2215 | 0.2700 | 0.2493 |
| D+ID+D | 0.2217 | 0.2701 | 0.2492 |
| D+ID+D | 0.2175 | 0.3074 | 0.2458 |
| sham2 | 0.1306 | 0.1252 | 0.1177 |
| sham2 | 0.1573 | 0.1287 | 0.1071 |
| sham2 | 0.1170 | 0.1136 | 0.1121 |
| sham2 | 0.1240 | 0.1216 | 0.1122 |
| Group | SFR 30 days after IR （ml/10min) | SFR 60 days after IR （ml/10min) | SFR 90 days after IR （ml/10min) |
| sham2 | 0.0910 | 0.1191 | 0.1117 |
| IR+D | 0.1756 | 0.2338 | 0.2334 |
| IR+D | 0.2166 | 0.2416 | 0.2337 |
| IR+D | 0.2011 | 0.2583 | 0.2354 |
| IR+D | 0.1882 | 0.2323 | 0.2223 |
| IR+D | 0.2161 | 0.2551 | 0.2238 |
| IR+D | 0.2012 | 0.2201 | 0.2461 |
| IR+D | 0.2496 | 0.2581 | 0.2214 |
| IR+D | 0.2090 | 0.2457 | 0.2309 |
| IR+D | 0.2171 | 0.2610 | 0.2156 |
| IR+D | 0.2146 | 0.2511 | 0.2467 |
| sham3 | 0.1475 | 0.1160 | 0.1205 |
| sham3 | 0.1338 | 0.1112 | 0.1092 |
| sham3 | 0.1202 | 0.1041 | 0.1254 |
| sham3 | 0.1115 | 0.1336 | 0.1176 |
| sham3 | 0.0679 | 0.1161 | 0.1158 |
| IR | 0.1190 | 0.1258 | 0.1185 |
| IR | 0.1404 | 0.1282 | 0.1194 |
| IR | 0.1263 | 0.1130 | 0.1269 |
| IR | 0.1241 | 0.1193 | 0.1196 |
| IR | 0.1105 | 0.1102 | 0.1136 |
