# Supplementary material for: Restoring the Secretory Function of Irradiation-Damaged Salivary Gland by Administrating Deferoxamine in Mice
Source: PLoS One. 2014 Nov 26;9(11):e113721. doi: 10.1371/journal.pone.0113721 (PMC4245233; doi:10.1371/journal.pone.0113721)
Supplement: Table S2 — The weight of each salivary gland harvested at 90th day post irradiation. Sham1: Pre-sterilized water group; sham2: Pre+Post sterilized water group; sham3: Post-sterilized water group. (DOC) [file pone.0113721.s002.doc]

**Table S2: The weight of each salivary gland harvested at 90^th^ day post irradiation. Sham1: Pre-sterilized water group; sham2: Pre+Post sterilized water group; sham3: Post-sterilized water group.**

| Group | the weight of SG(g) |
| --- | --- |
| Normal | 0.1989 |
| Normal | 0.1701 |
| Normal | 0.1908 |
| Normal | 0.1705 |
| Normal | 0.2088 |
| D+IR | 0.1540 |
| D+IR | 0.1536 |
| D+IR | 0.1520 |
| D+IR | 0.1556 |
| D+IR | 0.1501 |
| D+IR | 0.1588 |
| D+IR | 0.1575 |
| D+IR | 0.1448 |
| D+IR | 0.1501 |
| D+IR | 0.1615 |
| sham1 | 0.1509 |
| sham1 | 0.1510 |
| sham1 | 0.1465 |
| sham1 | 0.1506 |
| sham1 | 0.1467 |
| D+ID+D | 0.1564 |
| D+ID+D | 0.1605 |
| D+ID+D | 0.1604 |
| D+ID+D | 0.1526 |
| D+ID+D | 0.1520 |
| D+ID+D | 0.1610 |
| D+ID+D | 0.1539 |
| D+ID+D | 0.1437 |
| D+ID+D | 0.1602 |
| D+ID+D | 0.1600 |
| sham2 | 0.1520 |
| sham2 | 0.1531 |
| sham2 | 0.1510 |
| sham2 | 0.1449 |
| sham2 | 0.1444 |
| IR+D | 0.1561 |
| IR+D | 0.1426 |
| Group | the weight of SG(g) |
| IR+D | 0.1623 |
| IR+D | 0.1569 |
| IR+D | 0.1574 |
| IR+D | 0.1555 |
| IR+D | 0.1560 |
| IR+D | 0.1601 |
| IR+D | 0.1611 |
| IR+D | 0.1576 |
| sham3 | 0.1554 |
| sham3 | 0.1510 |
| sham3 | 0.1443 |
| sham3 | 0.1511 |
| sham3 | 0.1520 |
| IR | 0.1575 |
| IR | 0.1616 |
| IR | 0.1422 |
| IR | 0.1399 |
| IR | 0.1503 |
