# Supplementary material for: Restoring the Secretory Function of Irradiation-Damaged Salivary Gland by Administrating Deferoxamine in Mice
Source: PLoS One. 2014 Nov 26;9(11):e113721. doi: 10.1371/journal.pone.0113721 (PMC4245233; doi:10.1371/journal.pone.0113721)
Supplement: Table S3 — Surface area occupied by acinar cells (% per gland) of each salivary gland. Sham1: Pre-sterilized water group; sham2: Pre+Post sterilized water group; sham3: Post-sterilized water group. The software Image-Pro Plus 6.0 was used to analyze the surface area occupied by acinar cells. (DOC) [file pone.0113721.s003.doc]

**Table S3: Surface area occupied by acinar cells (% per gland) of each salivary gland.** Sham1: Pre-sterilized water group; sham2: Pre+Post sterilized water group; sham3: Post-sterilized water group. The software Image-Pro Plus 6.0 was used to analyze the surface area occupied by acinar cells.

| Group | surface area occupied by acinar cells(% per gland) |
| --- | --- |
| Normal | 65.4 |
| Normal | 67.33 |
| Normal | 66.21 |
| Normal | 66.47 |
| Normal | 67.56 |
| D+IR | 41.2 |
| D+IR | 40.22 |
| D+IR | 41.36 |
| D+IR | 39.74 |
| D+IR | 41.57 |
| D+IR | 39.85 |
| D+IR | 38.96 |
| D+IR | 40.27 |
| D+IR | 39.98 |
| D+IR | 40.2 |
| sham1 | 11.31 |
| sham1 | 11.2 |
| sham1 | 10.57 |
| sham1 | 10.33 |
| sham1 | 10.85 |
| D+ID+D | 55.1 |
| D+ID+D | 55.23 |
| D+ID+D | 56.41 |
| D+ID+D | 55.32 |
| D+ID+D | 54.57 |
| D+ID+D | 57 |
| D+ID+D | 53.71 |
| D+ID+D | 55.74 |
| D+ID+D | 55.37 |
| D+ID+D | 55.39 |
| sham2 | 10.54 |
| sham2 | 11.24 |
| sham2 | 10.56 |
| sham2 | 11.1 |
| Group | surface area occupied by acinar cells(% per gland) |
| sham2 | 10.86 |
| IR+D | 40.11 |
| IR+D | 39.54 |
| IR+D | 40.23 |
| IR+D | 41.1 |
| IR+D | 40.11 |
| IR+D | 40.21 |
| IR+D | 40.12 |
| IR+D | 39.66 |
| IR+D | 39.85 |
| IR+D | 40.1 |
| sham3 | 10.69 |
| sham3 | 10.86 |
| sham3 | 10.96 |
| sham3 | 11 |
| sham3 | 10.87 |
| IR | 10.76 |
| IR | 10.83 |
| IR | 10.91 |
| IR | 11.2 |
| IR | 10.93 |
