# Supplementary material for: Restoring the Secretory Function of Irradiation-Damaged Salivary Gland by Administrating Deferoxamine in Mice
Source: PLoS One. 2014 Nov 26;9(11):e113721. doi: 10.1371/journal.pone.0113721 (PMC4245233; doi:10.1371/journal.pone.0113721)
Supplement: Table S4 — Ratio of TUNEL-positive cells to total nuclei (% per gland) of each salivary gland. Sham1:Pre-sterilized water group; sham2: Pre+Post sterilized water group; sham3: Post-sterilized water group. The software Image-Pro Plus 6.0 was used to analyze the ratio of TUNEL-positive cells to total nuclei. (DOC) [file pone.0113721.s004.doc]

**Table S4:Ratio of TUNEL-positive cells to total nuclei（% per gland) of each salivary gland.** Sham1: Pre-sterilized water group; sham2: Pre+Post sterilized water group; sham3: Post-sterilized water group. The software Image-Pro Plus 6.0 was used to analyze the ratio of TUNEL-positive cells to total nuclei.

| Group | Ratio of TUNEL positive cells to total nuclei(% per gland) |
| --- | --- |
| Normal | 4.875 |
| Normal | 4.913 |
| Normal | 4.866 |
| Normal | 4.769 |
| Normal | 4.977 |
| D+IR | 12.45 |
| D+IR | 12.35 |
| D+IR | 12.561 |
| D+IR | 11.951 |
| D+IR | 12.411 |
| D+IR | 12.11 |
| D+IR | 12.391 |
| D+IR | 12.384 |
| D+IR | 12.3 |
| D+IR | 12.241 |
| sham1 | 24.309 |
| sham1 | 24.476 |
| sham1 | 24.298 |
| sham1 | 24.46 |
| sham1 | 24.333 |
| D+ID+D | 10.31 |
| D+ID+D | 10.27 |
| D+ID+D | 9.95 |
| D+ID+D | 10.09 |
| D+ID+D | 10.21 |
| D+ID+D | 9.85 |
| D+ID+D | 9.848 |
| D+ID+D | 10.34 |
| D+ID+D | 10.123 |
| D+ID+D | 10.984 |
| sham2 | 24.32 |
| sham2 | 24.487 |
| sham2 | 24.31 |
| sham2 | 24.6 |
| Group | Ratio of TUNEL positive cells to total nuclei(% per gland) |
| sham2 | 24.576 |
| IR+D | 13.55 |
| IR+D | 13.62 |
| IR+D | 13.47 |
| IR+D | 13.671 |
| IR+D | 13.57 |
| IR+D | 13.64 |
| IR+D | 13.49 |
| IR+D | 13.71 |
| IR+D | 13.56 |
| IR+D | 13.486 |
| sham3 | 24.32 |
| sham3 | 24.46 |
| sham3 | 24.33 |
| sham3 | 24.31 |
| sham3 | 24.5 |
| IR | 24.31 |
| IR | 24.35 |
| IR | 24.4 |
| IR | 24.41 |
| IR | 24.356 |
