# Supplementary material for: Restoring the Secretory Function of Irradiation-Damaged Salivary Gland by Administrating Deferoxamine in Mice
Source: PLoS One. 2014 Nov 26;9(11):e113721. doi: 10.1371/journal.pone.0113721 (PMC4245233; doi:10.1371/journal.pone.0113721)
Supplement: Table S5 — Surface area occupied by PCNA-positive cells (% per gland) of each salivary gland. Sham1: Pre-sterilized water group; sham2: Pre+Post sterilized water group; sham3: Post-sterilized water group. The software Image-Pro Plus 6.0 was used to analyze the surface area occupied by PCNA+ cells. (DOC) [file pone.0113721.s005.doc]

**Table S5: Surface area occupied by PCNA-positive cells (% per gland) of each salivary gland.** Sham1: Pre-sterilized water group; sham2: Pre+Post sterilized water group; sham3: Post-sterilized water group. The software Image-Pro Plus 6.0 was used to analyze the surface area occupied by PCNA^+^ cells.

| Group | surface area occupied by PCNA^+^ cells(% per gland) |
| --- | --- |
| Normal | 85.23 |
| Normal | 86.21 |
| Normal | 87.41 |
| Normal | 84.33 |
| Normal | 81.32 |
| D+IR | 38.66 |
| D+IR | 40.52 |
| D+IR | 37.85 |
| D+IR | 38.45 |
| D+IR | 39.1 |
| D+IR | 38.44 |
| D+IR | 38.84 |
| D+IR | 38.77 |
| D+IR | 38.82 |
| D+IR | 38.95 |
| sham1 | 10.55 |
| sham1 | 11.22 |
| sham1 | 11.4 |
| sham1 | 11.2 |
| sham1 | 11.09 |
| D+ID+D | 56.33 |
| D+ID+D | 55.77 |
| D+ID+D | 54.89 |
| D+ID+D | 54.99 |
| D+ID+D | 55.71 |
| D+ID+D | 56.1 |
| D+ID+D | 56.22 |
| D+ID+D | 57.1 |
| D+ID+D | 55.89 |
| D+ID+D | 55.87 |
| sham2 | 10.6 |
| sham2 | 11.1 |
| sham2 | 11.11 |
| sham2 | 10.51 |
| Group | surface area occupied by PCNA^+^ cells(% per gland) |
| sham2 | 10.83 |
| IR+D | 41.21 |
| IR+D | 41.24 |
| IR+D | 39 |
| IR+D | 38.55 |
| IR+D | 38.41 |
| IR+D | 37.88 |
| IR+D | 39.65 |
| IR+D | 38.31 |
| IR+D | 40.33 |
| IR+D | 39.39 |
| sham3 | 11.21 |
| sham3 | 10.44 |
| sham3 | 11.24 |
| sham3 | 10.8 |
| sham3 | 11.45 |
| IR | 11.23 |
| IR | 11.24 |
| IR | 10.55 |
| IR | 11.2 |
| IR | 11.06 |
