# Supplementary material for: Restoring the Secretory Function of Irradiation-Damaged Salivary Gland by Administrating Deferoxamine in Mice
Source: PLoS One. 2014 Nov 26;9(11):e113721. doi: 10.1371/journal.pone.0113721 (PMC4245233; doi:10.1371/journal.pone.0113721)
Supplement: Table S6 — Surface area occupied by CD31+ cells (% per gland) of each salivary gland. Sham1: Pre-sterilized water group; sham2: Pre+Post sterilized water group; sham3: Post-sterilized water group. The software Image-Pro Plus 6.0 was used to analyze the surface area occupied by CD31+ cells. (DOC) [file pone.0113721.s006.doc]

**Table S6: Surface area occupied by CD31**^+^ **cells (% per gland) of each salivary gland.**Sham1: Pre-sterilized water group; sham2: Pre+Post sterilized water group; sham3: Post-sterilized water group. The software Image-Pro Plus 6.0 was used to analyze the surface area occupied by CD31^+^ cells.

| Group | surface area occupied by CD31^+^ cells(% per gland) |
| --- | --- |
| Normal | 26.54 |
| Normal | 25.68 |
| Normal | 25.44 |
| Normal | 27.11 |
| Normal | 25.36 |
| D+IR | 16.77 |
| D+IR | 16.84 |
| D+IR | 17.12 |
| D+IR | 17.33 |
| D+IR | 16.89 |
| D+IR | 17.1 |
| D+IR | 17.01 |
| D+IR | 17.23 |
| D+IR | 16.78 |
| D+IR | 17.05 |
| sham1 | 5.41 |
| sham1 | 5.32 |
| sham1 | 5.24 |
| sham1 | 6.14 |
| sham1 | 5.53 |
| D+ID+D | 22.41 |
| D+ID+D | 21.14 |
| D+ID+D | 19.41 |
| D+ID+D | 19.56 |
| D+ID+D | 20.16 |
| D+ID+D | 19.89 |
| D+ID+D | 20.15 |
| D+ID+D | 19.54 |
| D+ID+D | 20.31 |
| D+ID+D | 20.29 |
| sham2 | 6.01 |
| sham2 | 5.14 |
| sham2 | 5.45 |
| sham2 | 5.61 |
| Group | surface area occupied by CD31^+^ cells(% per gland) |
| sham2 | 5.56 |
| IR+D | 18.24 |
| IR+D | 18.31 |
| IR+D | 18.41 |
| IR+D | 18.2 |
| IR+D | 17.85 |
| IR+D | 17.89 |
| IR+D | 18.44 |
| IR+D | 18.61 |
| IR+D | 19.11 |
| IR+D | 18.34 |
| sham3 | 5.41 |
| sham3 | 5.31 |
| sham3 | 5.61 |
| sham3 | 5.29 |
| sham3 | 5.38 |
| IR | 5.43 |
| IR | 5.43 |
| IR | 5.17 |
| IR | 5.67 |
| IR | 5.44 |
