# Supplementary material for: Restoring the Secretory Function of Irradiation-Damaged Salivary Gland by Administrating Deferoxamine in Mice
Source: PLoS One. 2014 Nov 26;9(11):e113721. doi: 10.1371/journal.pone.0113721 (PMC4245233; doi:10.1371/journal.pone.0113721)
Supplement: Table S7 — Real-time PCR detection of VEGF expression and Western-blot analysis of HIF-1α 90 days after irradiation. We showed the value of ΔCT of VEGF and HIF-1α densitometry in each group. Sham1: Pre-sterilized water group; sham2: Pre+Post sterilized water group; sham3: Post-sterilized water group. (DOC) [file pone.0113721.s007.doc]

Table S7:Real-time PCR detection of VEGF expression and Western-blot analysis of HIF-1α 90 days after irradiation. We showed the value of △ CT of VEGF and HIF-1α densitometry in each group. Sham1: Pre-sterilized water group; sham2: Pre+Post sterilized water group; sham3: Post-sterilized water group.

| Group | VEGF △ CT | HIF-1α densitometry |
| --- | --- | --- |
| Normal | 2.8 | 1.55 |
|  | 2.9 | 1.48 |
|  | 2.87 | 1.51 |
| D+IR | 6.01 | 0.6 |
|  | 5.85 | 0.58 |
|  | 6.02 | 0.61 |
| sham1 | 7.84 | 0.32 |
|  | 6.71 | 0.3 |
|  | 6.77 | 0.29 |
| D+IR+D | 3.06 | 1.04 |
|  | 3.4 | 1.03 |
|  | 3.36 | 1.01 |
| sham2 | 10.22 | 0.29 |
|  | 10.01 | 0.31 |
|  | 10.25 | 0.3 |
| IR+D | 5.05 | 1 |
|  | 5.11 | 0.97 |
|  | 5.67 | 0.99 |
| sham3 | 9.21 | 0.31 |
|  | 9 | 0.3 |
|  | 8.81 | 0.29 |
| IR | 7.51 | 0.27 |
|  | 6.79 | 0.28 |
|  | 6.93 | 0.29 |
